# Myocardial T1-mapping for early detection of left ventricular myocardial fibrosis in systemic sclerosis

**DOI:** 10.1186/1532-429X-13-S1-M10

**Published:** 2011-02-02

**Authors:** Franck Thuny, Leila Potton, Stanislas Rapacchi, Hélène Thibault, Cyrille Bergerot, Magalie Viallon, Nathan Mewton, Michel Ovize, Geneviève Derumeaux, Pierre Croisille

**Affiliations:** 1 Hopital Louis Pradel-Laboratoire Creatis-LRMN-UMR CNRS 5220 & INSERM U630, Lyon, France; 2 CHU de Grenoble, Grenoble, France; 3 Laboratoire Creatis-LRMN-UMR CNRS 5220 & INSERM U630, Lyon, France; 4 Hopital Louis Pradel, Lyon, France; 5 University Hospitals of Geneva, Geneve, Switzerland

## Background

Subclinical primary left ventricular (LV) dysfunction is a complication of systemic sclerosis (SSc) related to progressive diffuse myocardial fibrosis. Myocardial T1-mapping has recently been proposed as a cardiac magnetic resonance (CMR) method to quantify interstitial fibrosis early in the disease course whereas late-gadolinium enhancement (LGE) imaging remains normal.

## Objective

We aimed to evaluate whether myocardial T1-mapping could detect subclinical abnormalities in SSc patients with normal standard parameters of LV function and normal LGE imaging.

## Methods

We prospectively studied 37 patients (52±12 years) presenting with SSc with no history of heart disease, no pulmonary hypertension at rest, a normal LV assessed by conventional echocardiography (normal volumes, ejection fraction and wall motion), and no LGE. SSc patients were compared to16 matched controls healthy volunteers (47±7 years old). T1 quantification was performed using a Modified Look-Locker Inversion-recovery (MOLLI) sequence at 1.5T (Siemens) on a LV short axis before, 5min and 15 min after 0.2 mmol/Kg gadolinium injection. Imaging protocol included also standard Cine-SSFP imaging, and LGE imaging. LV diastolic function (mitral inflow pattern) was further assessed using echocardiography.

## Results

A non significant shorter mean T1 time (ms, mean±SEM) was observed in SSc patients compared to controls both at 5 (357±5 vs. 361±6, p=0.65) and 15 (448±5 vs. 456±5, p=0.34) minutes after gadolinium injection. Echocardiography displayed a LV diastolic dysfunction in 47% of SSc patients and in 25% of controls (p=0.04). The SSc patients with a LV diastolic dysfunction had a shorter 15 minutes post-contrast T1 time (ms) than those with a normal diastolic function (431±7 vs. 464±8, p=0.01).

## Conclusions

Post-contrast T1-mapping identifies changes in LV myocardial T1 times in patients with SSc, normal LVEF and impaired LV diastolic function. These abnormalities may reflect the impact of diffuse interstitial myocardial fibrosis in SSc that could be early detected in the future.

